# Supplementary material for: Transport Characteristics of Fujifilm Ion-Exchange Membranes as Compared to Homogeneous Membranes АМХ and СМХ and to Heterogeneous Membranes MK-40 and MA-41
Source: Membranes (Basel). 2019 Jul 14;9(7):84. doi: 10.3390/membranes9070084 (PMC6680501; doi:10.3390/membranes9070084)
Supplement: Supplementary file 1 [file membranes-09-00084-s001.pdf]

membranes

Supplementary Materials: Transport Characteristics of Fujifilm Ion-Exchange Membranes as Compared to Homogeneous Membranes AMX and CMX and to Heterogeneous Membranes MK-40 and MA-41

Veronika Sarapulova [1], Inna Shkorkina [1], Semyon Mareev [1], Natalia Pismenskaya [1,*], Natalia Kononenko [1], Christian Larchet [2], Lasaad Dammak [2] and Victor Nikonenko [1]

[1] Kuban State University, 149 Stavropolskaya st., 350040 Krasnodar, Russia
[2] Institut de Chimie et des Matériaux Paris-Est, UMR7182 CNRS—Université Paris-Est, 2 rue Henri Dunant, 94320 Thiais, France
* Correspondence: n_pismen@mail.ru; Tel.: +7-918-48-91-292

The total exchange capacity (Q_{sw}) is determined by the static acid-basic method [60]. At first, membrane samples were transformed into the OH^- (AEM) / H^+ (CEM) form and washed in distilled water. Prepared samples of the swollen membrane weighing about 2.0 g (m_{sw}), finely cut into pieces and put into conical flasks. In the case of AEM, these flasks were filled with a sodium hydroxide solution with a volume of 100.00 cm^3 with a concentration of 0.10 mol dm^{-3}. In the case of CEM, the same amount of sodium hydroxide solution with the same concentration was added. Then, the membranes were kept until equilibrium (for 24 h) in the above mentioned solutions periodically shaking. After that, an aliquot of the solution over membrane (25.00 cm^3) was taken and titration of 0.10 mol dm^{-3} with NaOH (AEM) or HCl (CEM) was carried out in the presence of a mixed indicator (3–5 drops). Titration was performed using EasyPlusTitrators (METTLER TOLEDO), with the output of the titration results data to a computer.

The calculation of the membrane total exchange capacity, Q_{sw} (mmol/g$_{sw}$), was carried out by equation:

$$Q_{sw} = \frac{(100 - 4V_1)}{10\ m_{sw}}$$

where V_1 is the volume of 0.10 M HCl solution (for strongly and weakly acidic cation-exchange membranes) or NaOH (for strongly and weakly basic anion-exchange membranes), spent on titration (mL); m_{sw} is the mass of a sample (g).

The calculation of the total exchange capacity of ion-exchange membranes in mmol / g$_{dry}$ (Q_{dry}) was carried out taking into account the water content of the membrane W:

$$Q_{dry} = \frac{(100 - 4V_1)}{10\ m_{sw}(1 - W)} \quad (1)$$

Water content (W, %) of the membranes is determined by the method of air-heat drying. Before the experiment, the samples were equilibrated with 0.02 mol dm^{-3} electrolyte solution at 25 ± 1 °C for 24 h. After equilibration, samples were taken out from the solution and the film of liquid was removed from the samples' ends and surfaces using filter paper.

Weights of wet, m_{sw}, and dry, m_{dry}, samples were obtained using an MB25 Ohaus moisture analyzer. The evaporation of water was carried out at a temperature of 50 °C to a constant weight of the sample.

The water content *W*, % was calculated by the formula:

$$W = \frac{m_{sw} - m_{dry}}{m_{sw}} \times 100\%$$

Some results of optical microscopy and XRF

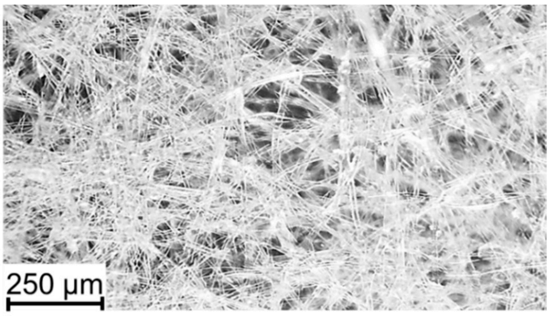

(**a**)

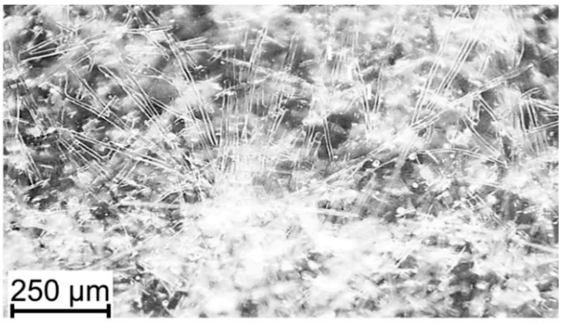

(**b**)

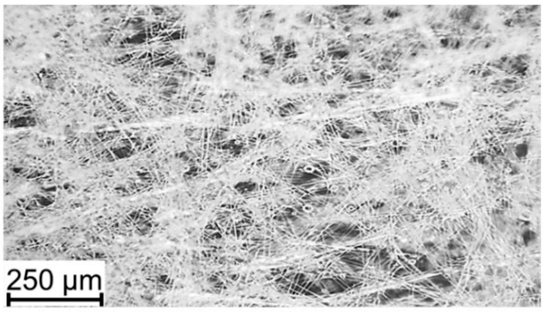

(**c**)

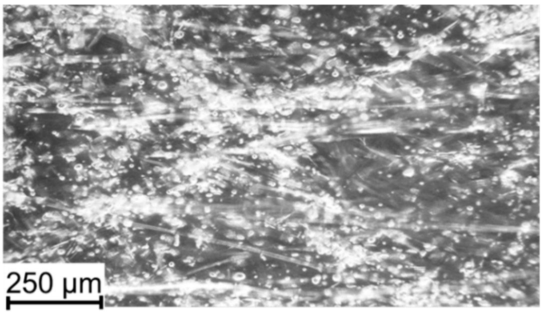

(**d**)

Figure S1. Optical images of air-dry AMX Type I (**a**), AMX Type II (**b**), CMX Type I (**c**), CMX Type II (**d**) membrane samples.

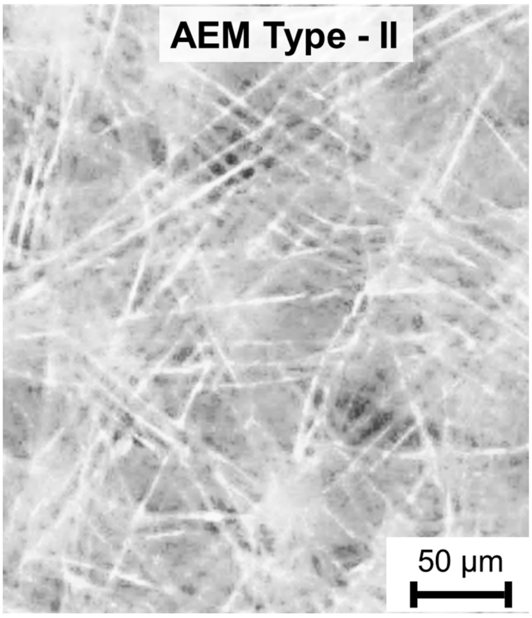

(**a**)

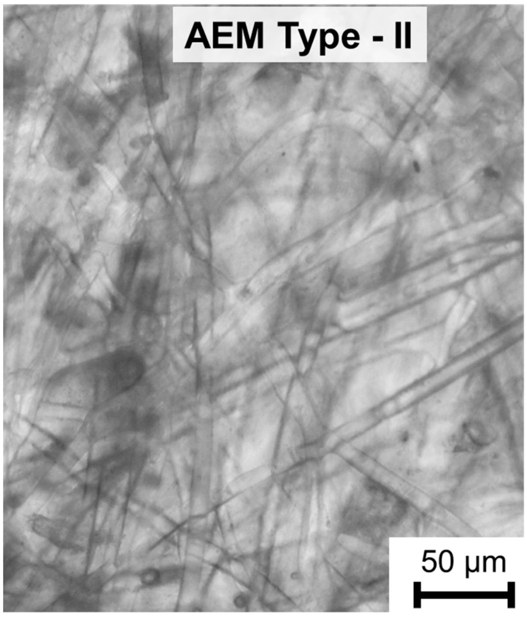

(**b**)

Figure S2. Optical images of the swollen AEM Type-II membrane. Samples soaked in a 0.02 M NaCl solution for 24 h (**a**), and the same samples in the 0.02 M NaCl solution where a high molecular weight dye (anthocyanin) is added (**b**).

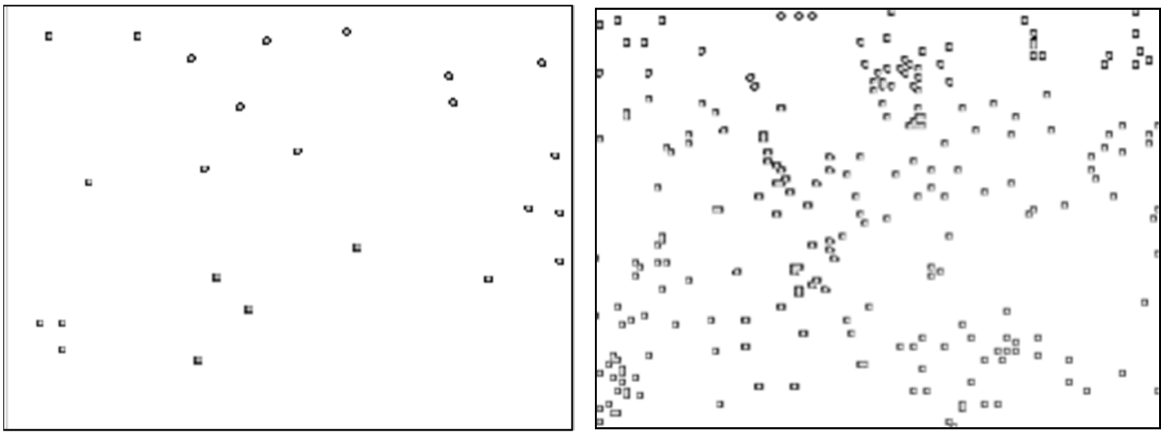

(**a**) (**b**)

Figure S3. The distribution of Cu^{2+} ions in the composition of complexes with secondary and tertiary amines in the cross-sections of AEM Type-X (**a**) and AEM Type-I (**b**) membranes. The area mapped using XRF is 100 × 100 μm^2.

© 2019 by the authors. Submitted for possible open access publication under the terms and conditions of the Creative Commons Attribution (CC BY) license (http://creativecommons.org/licenses/by/4.0/).
